# Supplementary figures and images for: Accelerated coronary MRI with sRAKI: A database-free self-consistent neural network k-space reconstruction for arbitrary undersampling
Source: PLoS One. 2020 Feb 21;15(2):e0229418. doi: 10.1371/journal.pone.0229418 (PMC7034900; doi:10.1371/journal.pone.0229418)

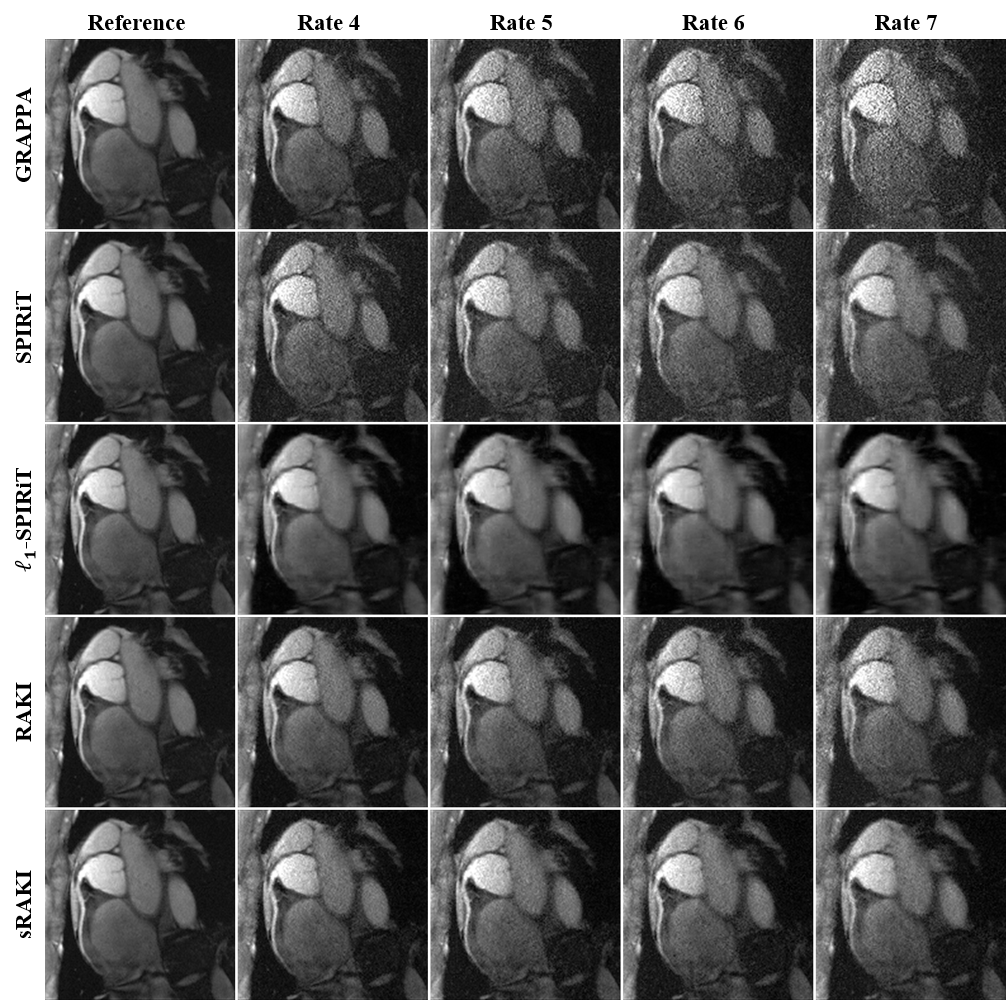

Supplement: S1 Fig — The data were uniformly undersampled retrospectively at rates 2×2, 3×2, 4×2 and 5×2 in ky−kz plane, which are approximately equivalent to net acceleration rates 4, 5, 6 and 7 (including the ACS lines and an elliptical mask). These data were then reconstructed using GRAPPA, SPIRiT, l1-SPIRiT, RAKI and sRAKI (from top to bottom). Acceleration rate was set no higher than 2 for kz dimension, since the size of data along this dimension was small (20 lines in total and 10 lines for ACS). For RAKI, a 3-layer network was designed with a kernel size of 2×2 (with dilations equaling acceleration rates to match the undersampled uniform pattern) for the first layer and a kernel size of 1×1 for subsequent layers. Note that this 2D undersampling is different from the original RAKI paper, thus the network architecture may be sub-optimal. The learning rate and number of epochs for RAKI were tuned to 0.05 and 2000 iterations, respectively. Fully-sampled images are also displayed in the first column as a reference for comparison. While RAKI is robust, GRAPPA is very sensitive to noise with increasing rates. In addition, RAKI outperforms SPIRiT, but RAKI and sRAKI perform comparatively, similar to the relationship between GRAPPA and SPIRiT. (TIF) [file pone.0229418.s001.tif]

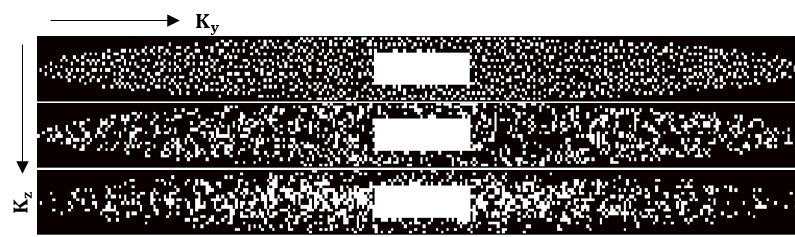

Supplement: S2 Fig — Poisson disc (top), uniform-density random (middle) and variable-density random (bottom) with 4-fold acceleration. (TIF) [file pone.0229418.s002.tif]

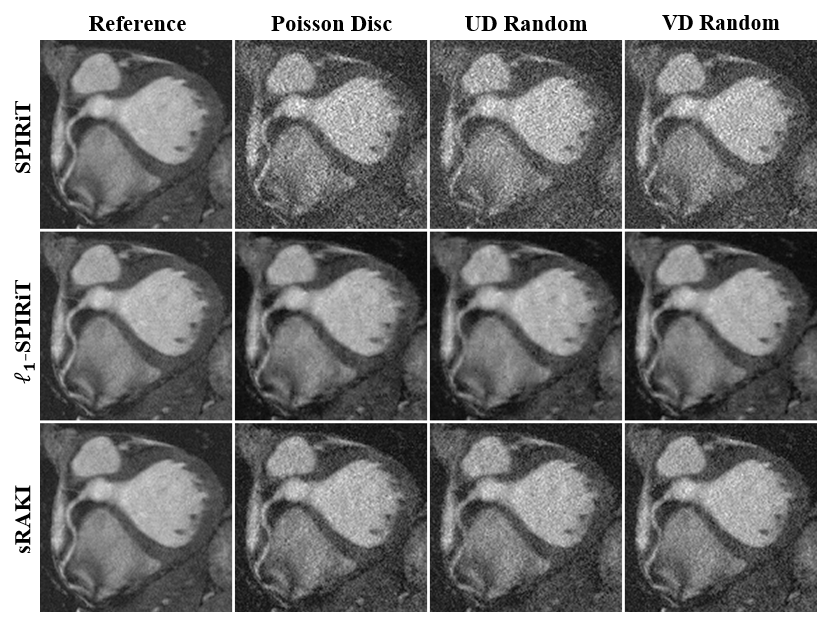

Supplement: S3 Fig — The data were retrospectively undersampled with the three different patterns shown in S2 Fig. These data were then reconstructed using SPIRiT, l1-SPIRiT and sRAKI. The results show that sRAKI is more resilient to noise amplification compared with SPIRiT, regardless of undersampling pattern. (TIF) [file pone.0229418.s003.tif]

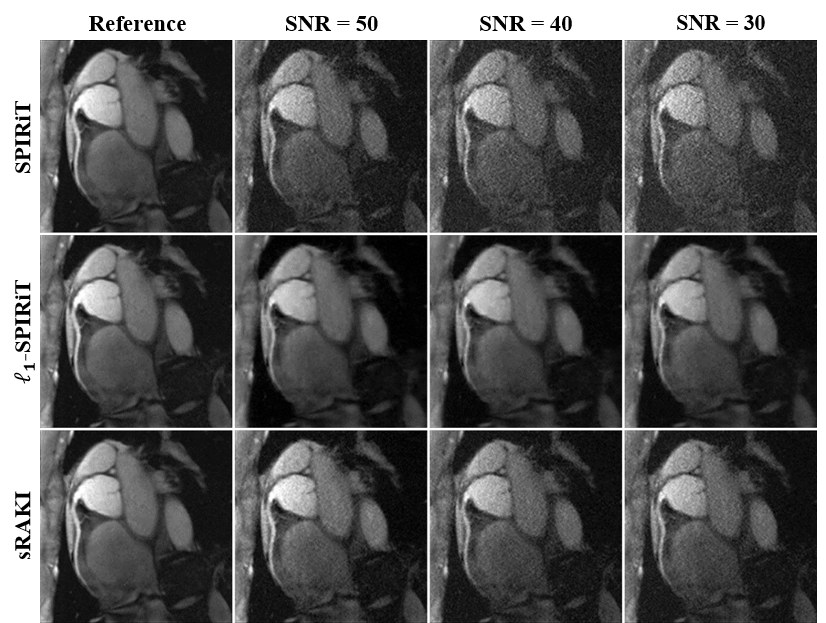

Supplement: S4 Fig — Additive Gaussian noise was added to the datasets retrospectively, and the reported SNR was measured at aorta (signal power at aorta divided by noise power in an empty region of interest), with the original dataset having SNR of 50. The datasets were then retrospectively undersampled at rate 4 and reconstructed using SPIRiT, l1-SPIRiT and sRAKI. sRAKI is more robust against noise of data compared with SPIRiT. However, noise amplification becomes evident with increasing levels of noise compared with l1-SPIRiT. (TIF) [file pone.0229418.s004.tif]

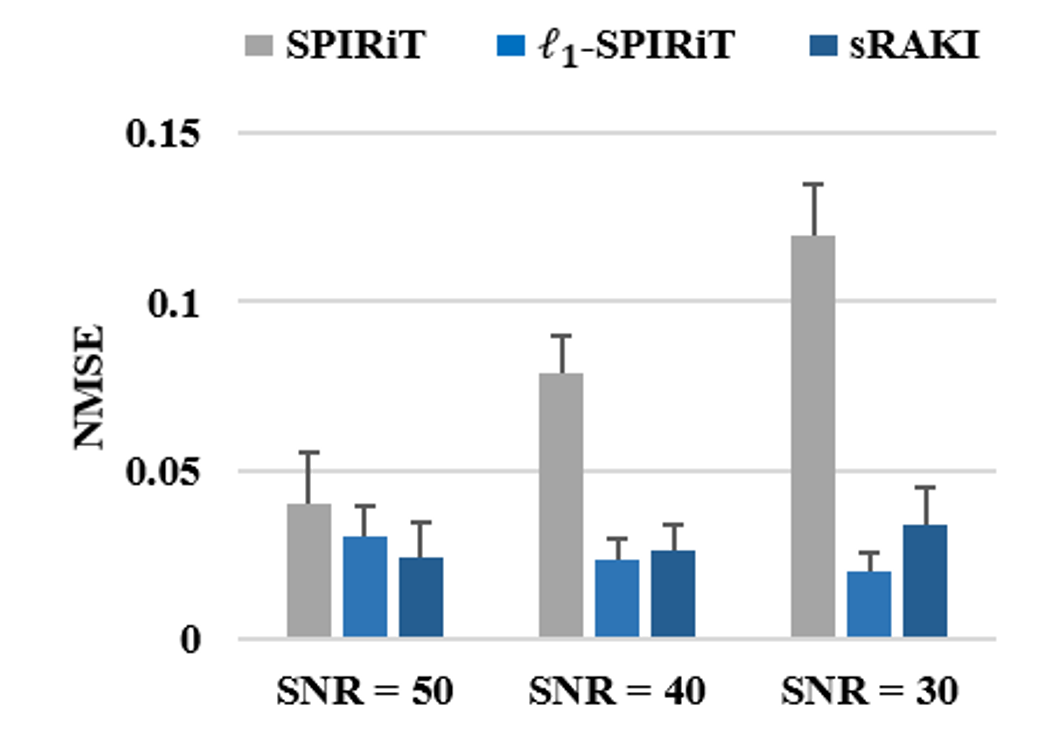

Supplement: S5 Fig — Error bars represent standard deviation across subjects. (TIF) [file pone.0229418.s005.tif]
